# Addressing COVID-19 Screening Delays: The Impact of HPV Self-Sampling on Non-Attenders in a Cervical Cancer Screening Program

**DOI:** 10.3390/cancers16234071

**Published:** 2024-12-05

**Authors:** Angela Chiereghin, Lorenzo Pizzi, Carolina Buriani, Tiziana Sanna, Andrea Amico, Lorena Squillace, Elena Molinari, Maria Siponta Florean, Giovanni Lanza, Francesca Mezzetti

**Affiliations:** 1 Governance of Screening Programs Unit, Local Health Authority of Bologna, 40124 Bologna, Italy; l.pizzi@ausl.bologna.it (L.P.); tiziana.sanna@ausl.bologna.it (T.S.); lorena.squillace@ausl.bologna.it (L.S.); elena.molinari@ausl.bologna.it (E.M.); m.florean@ausl.bologna.it (M.S.F.); francesca.mezzetti@ausl.bologna.it (F.M.); 2 Anatomic Pathology Unit, University Hospital of Ferrara, 44124 Ferrara, Italy; c.buriani@ospfe.it (C.B.); andrea.amico@ospfe.it (A.A.); 3 Anatomic Pathology Unit, Department of Translational Medicine, University of Ferrara, 44121 Ferrara, Italy; giovanni.lanza@unife.it

**Keywords:** organized cervical cancer screening program, self-sampling, HPV-related diseases

## Abstract

**Background/Objectives**: Self-sampling is recognized as a viable alternative to clinician-sampling for HPV primary screening. This study aimed to assess, within an Italian organized cervical cancer screening program, the acceptance and ease of use of self-sampling and the adherence to follow-up. The prevalences of HPV infection, cervical dysplasia, and cancer were contextually evaluated. **Methods**: Electronic records of 19,327 women, 30–64 years-old, residing within the Bologna Local Health Authority territory, who were offered self-sampling as an alternative to clinician-sampling, were retrospectively reviewed. They had never or irregularly attended and were overdue for a screening invitation due to the COVID-19 pandemic. An opt-in approach was adopted, involving local pharmacies for kit delivery and sample collection. Initially, HPV-positive results led to direct referral to colposcopy; later, cytological triage on clinician-samples was provided. **Results**: Self-sampling reached over twice as many women (11.5%) compared to historical clinician-sampling alone (<5%), showing high acceptance. Additionally, a high screening completion level was observed, with 79.5% of self-samples returned to pharmacies. A low percentage of self-samples resulted in inadequate results (1.1%), suggesting the method’s ease of use. HPV-positivity was 13.1%, higher than the 9.9% recorded in the ordinary screening population in 2019 (*p* < 0.001), the last year before the pandemic. Compliance to both immediate colposcopy and cytology triage exceeded 90% (*p* = 0.675). The rate of cervical adenocarcinoma was twice as high as in the routinely screened population in 2019 (0.9‰ versus 0.4‰). Finally, 6% of women opted for clinician appointments. **Conclusions**: Self-sampling proved to be an easy-to-use and effective tool for reaching non-attenders, who are at high risk of cancer. Cytology triage on clinician-samples did not negatively impact follow-up adherence. It seems appropriate to maintain a clinician-collection option even among non-attenders.

## Simple Summary

Self-sampling for HPV primary screening is recognized as a viable alternative to clinician-sampling. Its use in an Italian cervical cancer prevention program, focusing on acceptance, ease of use, and follow-up adherence, was evaluated. Self-sampling was offered to 19,327 women aged 30–64, overdue for screening due to the COVID-19 pandemic; they had never or irregularly attended. Results showed 11.5% of women chose self-sampling, more than doubling the participation rate compared to clinician-sampling alone (<5%), showing high acceptance. The return rate of self-samples was high (79.5%), with 1.1% being inadequate, indicating ease of use. HPV positivity was higher in the self-sampled group (13.1%) compared to the 2019 ordinary screening population (9.9%). Compliance with follow-up procedures exceeded 90%, and cervical cancer detection rates were higher (0.9‰) than in the routine screening population (0.4‰). Self-sampling was therefore effective in reaching non-attenders, but clinician-sampling remains important, as 6% of women opted for a clinician appointment.

## 1. Introduction

Currently, cervical cancer is almost completely preventable due to the availability of effective primary and secondary prevention measures, such as vaccination and screening. Nevertheless, globally in 2022, cervical cancer ranked fourth both in incidence and mortality [1].

It is well known that, in many countries, the COVID-19 (coronavirus disease 19) pandemic led to a temporary suspension of cervical cancer screening activities [2,3]. In Italy, the pandemic caused an average delay of 6 months in screening invitations [4]. In the UK, approximately 630 excess cancers were estimated over one screening round as consequence of 6 months’ delay [5]; however, it has been reported that a prompt restoration of cervical screening services appears to limit the impact [2]. In this regard, offering Human Papilloma Virus (HPV) self-sampling instead of in-clinic appointments to all women or to women in high-risk categories has been identified as an innovation for resuming routine cervical screening [6].

The literature reports that the self-sampling method can increase screening participation across countries with varying income levels, including low-, middle- and high-income nations [7,8]. Additionally, the Word Health Organization has recommended that, in settings where HPV testing is available, programs should consider whether the inclusion of HPV self-sampling as a complementary option within their existing approaches to cervical screening could address gaps in current coverage [9]. HPV self-sampling has recently been introduced as a primary screening method in routine screenings [10]. Some countries, such as Australia, Malaysia, and Denmark, offer HPV self-sampling to women who do not attend screenings. Other countries, like Argentina, Sweden, and the Netherlands, offer HPV self-sampling as an alternative for women who prefer not to undergo clinician-collected cervical smears [10,11].

From 2 December 2021 to 5 April 2022, the Local Health Authority (LHA) of Bologna, in the Emilia-Romagna Region (Northen Italy), offered the self-sampling method to 30–64 year-old women overdue for screening due to the COVID-19 pandemic as an alternative to booking a clinician appointment; the median delay in invitation was 8 months (range, 2–15 months). These women had either never or had irregularly attended the Bologna LHA’s organized screening program, i.e., never participated at all or participated in at least one round excluding the last, respectively.

The study primarily aimed to assess the acceptance and ease of use of the self-sampling method for HPV testing in this specific group of women, as well as to evaluate whether management through direct referral to colposcopy after a positive high-risk (hr)HPV-DNA result, compared to cytological triage on a clinician-collected sample, influenced follow-up adherence. Furthermore, the prevalence of HPV infection, cervical dysplasia, and cancer was evaluated in context. Few studies on this topic have been published so far in the setting of Italian organized cervical cancer screening programs [12,13,14,15,16].

Women in the target population who were due for screening examination during the study period were not offered self-sampling method but were invited to undergo clinician-collected screening.

## 2. Materials and Methods

### 2.1. Study Setting

In Italy, implementing population-based cervical screening programs has been recommended by national guidelines since 1996 [17] and has been included in the essential levels of assistance since 2001 [18]. Similarly to the national colorectal and breast cancer screening programs [19,20], the cervical cancer screening program’s administration is a collaborative effort between regions and LHAs, which in fact leads to some differences in regional strategies [21].

At the time of writing, the organized cervical screening program of the Bologna LHA, following the indications of the Emilia Romagna Region [22], uses the Pap test as the primary screening method for women aged 25–29 who have not received two doses of the HPV vaccine by age 15, and HPV DNA testing for women aged 30–64. HPV-vaccinated women with two doses of vaccine by 15 years of age are involved in cervical screening from the age of 30. For women aged 30–64, the shift from cytology to HPV testing occurred in April 2016 [23]. In the case of negative results, the Pap test and HPV test are offered every three and five years (screening round), respectively.

The overall target population of the Bologna LHA’s program is approximately 52,000 women per year.

### 2.2. Study Design

The study planned to retrospectively review electronic records of the women invited to participate at the first-level screening test for cervical cancer, with the option of using the self-sampling method. Women whose invitation letters were undelivered, who had moved out of the Bologna LHA area, or who were deceased were excluded from the study population.

The acceptance and ease of use of the self-sampling method were evaluated by considering the percentage of invited women who chose to attend screening using this optional method and the percentage of self-sampled vaginal samples resulting inadequate by hrHPV-DNA testing, respectively. The deadline for assessing the screening participation rate among eligible women was 31 December 2022, giving women a minimum of 9 months and a maximum of almost 13 months to attend screening. The participation rate for clinician-sampled screenings was also evaluated.

This retrospective study was approved by the Ethical Committee of the LHA of Bologna on 21 July 2022 (protocol code 503-2022-OSS-AUSLBO).

### 2.3. Local Procedures for Offering HPV Self-Sampling

A customized invitation letter was created for eligible women, informing them about the optional use of home-based HPV self-sampling. Additionally, a specific flyer was developed, outlining the steps to follow for participating in cervical screening using the self-sampling method.

**Figure 1:**
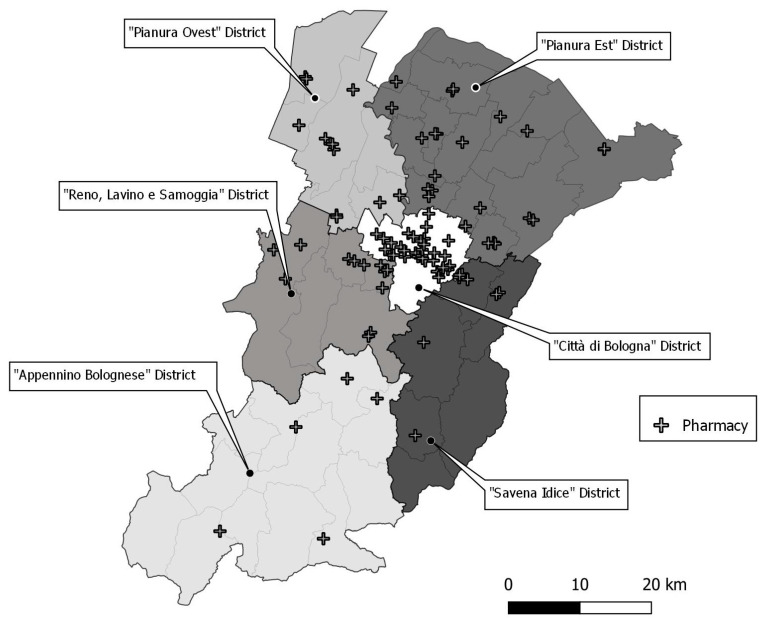
Distribution and number of local pharmacies participating in the cervical screening program by district of residence within the jurisdiction of the Bologna Local Health Authority.

An opt-in approach was adopted and, similarly to the organization of the colorectal screening program in the Bologna Screening Center, local pharmacies were involved as kit delivery points as well as sample collection and for sending samples to the laboratory. The pathway was governed by a contractual agreement, referred to as the “*Accordo tra Azienda USL di Bologna_Associazioni Farmacie*”, 2 December 2021. This organization ensures complete traceability of the kit-sample flow [19]. A total of 35.8% of private and public pharmacies (i.e., 92 out of 257) within the Bologna LHA territory were engaged. These pharmacies were selected based on the district of residence/domicile of eligible women and their annual workload in the colorectal cancer screening program. Specifically, a higher number of pharmacies were selected in the Città di Bologna District (Figure 1), where the majority (i.e., 57.9%) of eligible women resided. Additionally, pharmacies had to have supplied more than 350 occult blood test kits in the previous year of activity. Kit pick-up and self-sample returns could be performed at two different pharmacies, depending on the women’s convenience.

As previously reported [24], one and two months after the screening invitation, women received a text message reminder to pick up the self-sampling device at a local pharmacy for cervical screening. Additionally, women who had already picked up the device received two text message reminders, as well as a letter reminding them to collect the vaginal sample and return it to a local pharmacy, one, two, and three months after device pick-up, respectively [24].

Women who preferred to have a clinician-collected cervical smear could book a clinician appointment through one of the following routes: (i) the Screening Center’s online appointment portal, allowing citizens to autonomously schedule their appointments [25], (ii) written communication sent to the Screening Center’s email address, or (iii) a phone call to the Screening Center’s toll-free number.

### 2.4. Self-Collected Vaginal Swabs and hrHPV-DNA Testing

Self-collection was performed using dry, sterile, flocked swabs (Floqswab^®^, Copan SpA, Brescia, Italy) according to the manufacturer’s instructions. hrHPV-DNA testing was conducted at the laboratory associated with the screening program, specifically the Anatomic Pathology Unit at the University Hospital of Ferrara, Italy. Briefly, following the protocol previously described by Saville et al. [26], self-samples were eluted in 5 mL of ThinPrep PreservCyt^®^ media (Hologic Inc., Marlborough, MA, USA) upon arrival at the laboratory and analyzed using the Cobas^®^ 6800 System (Roche Diagnostics, Monza, Italy), in accordance with the manufacturer’s instructions. This molecular assay allows the detection of 12 hrHPV genotypes (31, 33, 35, 39, 45, 51, 52, 56, 58, 59, 66, and 68) (target channel 1; detected as a pool of other hrHPV genotypes), HPV16 (target channel 2), and HPV18 (target channel 3). Moreover, simultaneously, a fragment of the human β-globin gene was amplified during the reaction to ensure the presence and adequacy of cells. Samples were considered inadequate in the case of invalid results for one or more target combinations. For invalid target results, the original specimen was re-tested one time to obtain valid results. If the results were still invalid, a new specimen was required.

If the time elapsed between sample collection and arrival at the laboratory exceeded 14 days (i.e., the sample stability set by the self-collection device manufacturer), the self-collected flocked swabs were not investigated, and women were contacted by the LHA Screening Centre’s health staff to repeat the sampling; samples were stored and transported under a controlled temperature (+4 °C). Similarly, in the case of an inadequate sample, women were contacted by the LHA Screening Centre’s health staff and recommended cervical sampling by a healthcare worker, with an appointment offered.

### 2.5. Management of Women with hrHPV-DNA Positive Results

**Figure 2:**
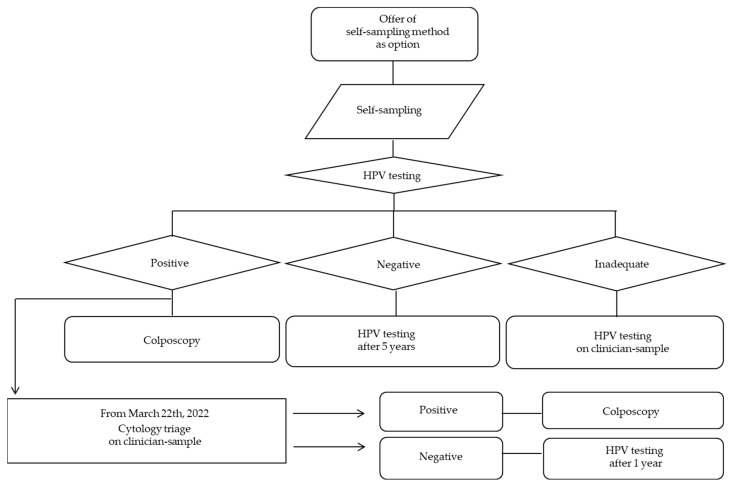
Flow-chart of the process.

The flow-chart of the process is illustrated in Figure 2.

Notably, after a positive hrHPV-DNA result, a direct referral to colposcopy policy was adopted during the first 3.7 months, followed by a cytology triage policy using clinician sample for the remaining months. This organizational change was prompted by a higher-than-expected number of hrHPV-DNA positive samples obtained.

Cytology findings were classified according to the Bethesda grading system 2014 [27]. Cervical colposcopy-directed biopsies were classified as normal; cervical intraepithelial neoplasia (CIN) grade 1, 2, 3; adenocarcinoma in situ; and invasive cancer according to international criteria [28].

### 2.6. Statistical Analysis

A univariate analysis was conducted using the χ^2^ test for all categorical variables and Student’s *t*-test for independent samples to compare continuous variables. To account for potential confounders, a multivariate logistic regression model was employed. A two-sided *p*-value < 0.05 was deemed statistically significant. The results from the multivariate logistic models were reported as Odds Ratios (OR) with 95% confidence intervals (95% CI) and corresponding *p*-values. Positive predictive values were determined based on the likelihood of having a CIN2+ lesion given a positive HPV test or Pap test.

The study results were analyzed in relation to data from the ordinary screening population recorded in 2019, considered a standard activity year before the COVID-19 pandemic emergency. In particular, the data of the Bologna LHA regarding the ordinary screening population refer to the calculation of indicators according to the national GISCi (Italian Cervicocarcinoma Screening Group) survey.

Statistical analysis was performed using Stata Statistical Software (Version 16.1).

## 3. Results

**Figure 3:**
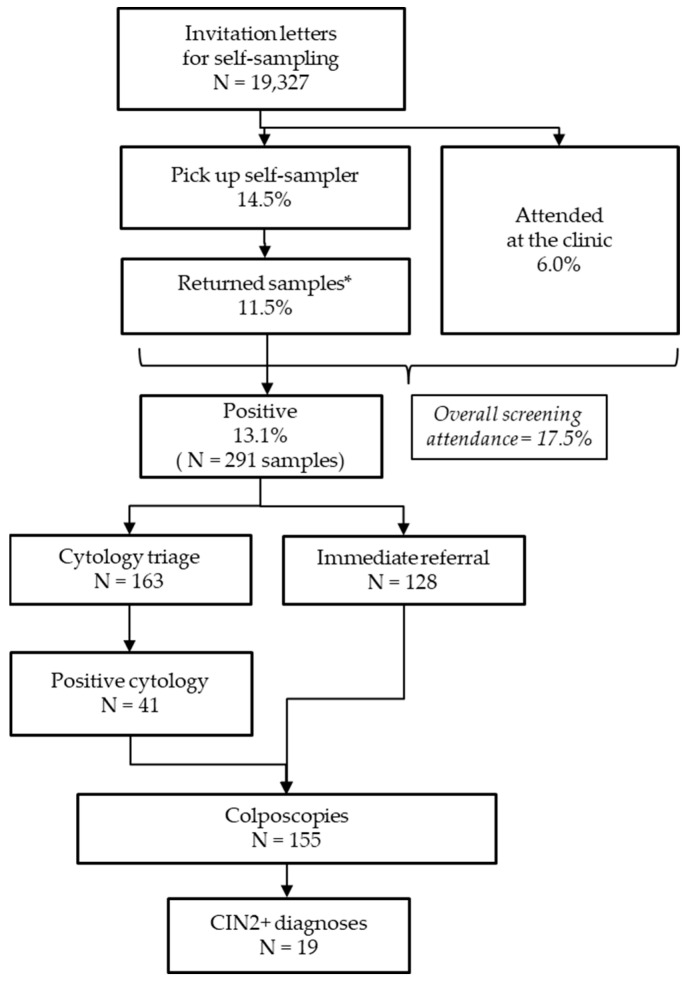
Results from the invitation for women’s screening to histological outcomes. * The eleven vaginal self-samples that were not investigated were not included.

The main results from the invitation for women’s screening to histological outcomes are depicted in Figure 3.

### 3.1. Characteristics of the Invited Women

The option to use the self-sampling method was offered to 19,327 women. Concurrently, 13,000 women were invited to participate in the “standard” screening program.

Among the women invited to use self-sampling method, 12,280 (63.5%) had never attended the organized cervical cancer screening program in Bologna, while 7047 (36.5%) had attended irregularly in the past. The latter group was categorized based on their last examination conducted as part of the organized screening program into women who had not been screened for less than 10 years and those who had not been screened for more than 10 years. On average, these women had gone without screening for about 11 years (minimum–maximum, 6–22 years).

The women’s demographic characteristics including age, nationality (Italian/Other), district of residence/domicile, and previous screening history are reported in Table 1.

The majority of women were under 40 years old (46.4%), Italian (75.2%), resided or were domiciled in the Città di Bologna District (57.9%), and had never attended the organized screening (63.5%) (Table 1). A total of 2% (n = 387) of the women had received three-dose HPV vaccination; the mean age at the time of the third dose was 33 years [SD ± 7.8].

Notably, based on their previous screening history, never attenders were classified as women with “no protection”. Women who had had their last screening test more than 10 years prior to the invitation for self-sampling were considered to have “low protection”, while those who had had their last screening test within the last 10 years were categorized as having “medium protection” [29].

### 3.2. Response to the Offer of Self-Sampling Method

Among the 19,237 invited women, 2799 (14.5%) picked up the swab for self-sampling at the pharmacy. Of these, 2226 women (2226/19,237; 11.5%) returned the self-sampled vaginal sample, resulting in a screening completion rate of 79.5% (2226/2799 women). Additionally, 1157 women (1157/19,237; 6.0%) chose to attend the clinic and booked a clinician appointment. The overall screening attendance in the study population was 17.5% (3383/19,237). The screening participation rate in the “standard” screening program was 60% (7800/13,000 women).

A statistically higher response rate to the alternative collection method was observed among women under 40 years of age, of Italian nationality, domiciled/resident in the Pianura Est District, and who had been underscreened for less than 10 years (15.7%) (Table 2).

Multivariate regression analysis results confirmed those of the univariate analysis regarding screening participation using the self-sampling method, with the exception of the district of residence, which was no longer found to be associated with the response to the optional offer of self-sampling (Table 3).

Finally, the incidence of HPV vaccination among women participating in screening with the self-sampling method was equal to 2.1% (47/2226).

### 3.3. Results of hrHPV Testing on Self-Collected Samples

With regard to the prevalence of HPV infection, 291 women (291/2226; 13.1%) tested positive for hrHPV-DNA. In particular, younger women had significantly higher HPV positivity rates than older women. HPV-DNA positivity was also significantly associated with previous screening history, with the highest rates observed in women who had never been screened, followed by those who had had their last screening examination more than 10 years before the invitation for self-sampling (Table 2). The HPV genotypes detected, along with information on HPV vaccination, are reported in Table 4.

Among the women who tested positive for hrHPV-DNA, 65.7% were positive for the pool of twelve other hrHPV genotypes, followed by 17.2% who tested positive for a mono-infection by HPV 16. Of these women, 4.1% (12/291) had been vaccinated (Table 4).

The overall percentage of inadequate self-collected samples was equal to 1.1% (24/2226 samples). The rate of technically inadequate self-collected samples was associated with women’s age, with the highest value observed among older women; it was not associated with nationality, district of residence, or previous screening history (Table 2).

The mean time between sample collection and arrival at the laboratory was 3 days. However, a total of 11 self-collected flocked swabs (11/2237; 0.5%) were not analyzed because this time frame exceeded the maximum allowable limit; the women were contacted by the LHA Screening Centre’s health staff and repeated the sampling at the clinic.

### 3.4. Compliance with a Follow-Up Examination After a Positive hrHPV Test and Histological Outcomes

Out of the 291 women who tested hrHPV-DNA positive from self-collected samples, 128 (44%) were directly referred to colposcopy, while 163 (56%) were recalled for cytology triage (Figure 3). Compliance rates for colposcopy and cytology triage were 92.2% (118/128 women) and 90.8% (148/163 women), respectively, with no significant difference between the two groups (*p* = 0.675). Among the women who underwent cytology triage, the Pap test was positive in 27.7% of cases (41/148 women), and compliance with subsequent colposcopy was 90.2% (37/41 women).

Among the 155 women who had a colposcopy, 19 (12.3%) were diagnosed with CIN2+ lesions, resulting in a detection rate of 8.5‰ (19/2226 screened women). The Positive Predictive Value (PPV) of HPV tests on self-collected samples and cytology triage for CIN2+ in women who underwent colposcopy is reported in Table 5. For this analysis, as carried out previously by Ivanus and colleagues [29], women with no/low protection were grouped together.

The majority (78.9%; 15/19 cases) of CIN2+ diagnoses were found in the group of women with “no/low protection”, including 4 out of 5 (80%) diagnoses of CIN3 and both (100%) diagnoses of cervical adenocarcinoma.

A total of 11% (11/100) of women with monoinfection by HPV16 or multi-infection involving HPV16 were diagnosed with CIN2+, compared to 4.2% (8/191) of women who tested positive for the pool of 12 other high-risk HPV genotypes (*p* = 0.025).

The CIN3+ lesions were associated in 71.4% (5/7) of cases with monoinfection by HPV16. None of the 12 HPV-vaccinated women were diagnosed with CIN3+.

Overall, a higher PPV was observed in the group of women recalled for cytology triage on clinician-sample compared to those immediately referred to colposcopy (16.2% versus 11.0%, respectively); however, the difference was not statistically significant (*p* = 0.400).

## 4. Discussion

Growing evidence supports the self-sampling method as a viable alternative to clinician-collected samples for primary HPV screening, particularly in efficiently reaching underscreened women [30]. Additionally, the adoption of HPV self-sampling was fast-tracked by the COVID-19 pandemic as it diminished the risk of virus exposure and extended healthcare services, helping to tackle delays in screening [30].

Here, we presented real-life data from a large Italian Screening Center that offered self-sampling as an alternative to clinician sampling for women aged 30–64 years who had either never or irregularly attended organized screening in the past. Self-sampling was offered to help re-establish the cervical cancer screening routine in response to the COVID-19 disruption. The response to the self-sampling method, the management of hrHPV-DNA positive women, the prevalence of HPV infection, as well as HPV-related cervical diseases were reported here.

Despite the literature suggesting that an opt-out approach is the most effective in terms of screening uptake [31], a HPV self-sampling option was offered locally which adopted an opt-in approach. This organizational decision was driven by a focus on minimizing resource waste, given that historical screening participation rates among never and irregular attenders at our center were below 5%. The choice to involve local pharmacies in this initiative originated from our previous positive assessment of their integration into colorectal cancer screening, which improved both service quality and screening attendance [32].

The response rates to offers of self-sampling methods have been found to be highly variable across different settings [31]. This high variability, influenced by factors such as the income levels of the countries, the approaches used for providing self-sampling kits, and the populations targeted, complicates comparisons. However, in our setting, offering the option of self-sampling for cervical cancer screening allowed us to reach a percentage of women more than twice as high (i.e., 11.5%) as the percentage historically achieved with the exclusive invitation to clinical sampling, showing positive acceptance by women. In addition, low vaginal swab waste was achieved, as high screening completion was observed among women who picked up a swab; nearly 80% of vaginal samples were returned to pharmacies. A positive effect of the interventions adopted to limit resource waste and promote screening participation was observed. In particular, 10.9% and 13.2% of women who received a text message reminder to collect the vaginal sample one and two months after picking up the swab, respectively, returned their samples to the pharmacy. A total of 24.2% of women who received a reminder letter for collecting the vaginal sample three months after picking up the swab returned their samples to the pharmacy [24]. Furthermore, as with the colorectal cancer screening program [32], the screening completion data suggest a positive acceptance among women of the role of pharmacies in distributing and collecting swabs and samples for cervical cancer screening. In line with other studies [29,33,34,35,36], the response rate to the offer of the self-sampling method was associated with women’s age and screening attendance history. In particular, multivariate regression analysis showed a higher response among younger women and those who had undergone a screening test within 10 years prior (i.e., women with medium protection); the lowest response rate was observed in never-screened women (i.e., women with no protection). A higher response was also observed among women of Italian nationality, who represented the majority (i.e., 75.2%) of the initiative’s target population as well as the ordinary screening population. Several initiatives are currently ongoing in our center to promote participation in screening among non-Italian women as part of equity-focused projects. For instance, direct interaction with trusted leaders of non-Italian communities was one of the strategies used for recruitment and engagement. Health education initiatives focused on enhancing screening awareness and discussing women’s perceptions about the HPV self-sampling method were carried out. Indeed, it has been reported that the acceptability of self-sampling highly depends on individual knowledge and beliefs, which is the reason why appropriate interventions are needed to maximize the effectiveness of this new screening strategy [15,37]. In this regard, women who pick-up a self-sampling swab from a local pharmacy receive a text message on their mobile phone informing them that there is a laboratory control checking the adequacy of the self-collected sample and that in the case of an inadequate sample they will be contacted by the health personnel of the Screening Center. This measure was implemented to enhance women’s confidence in the quality of the new strategy adopted. Finally, meetings were held with pharmacies involved in the cervical screening pathway and their Associations to provide information about the self-sampling method and how to effectively communicate with women about this new screening tool.

The fact that 6% of the invited women preferred to book a clinician appointment indicates that it is yet appropriate to offer both sample collection options for cervical screening to irregularly attending or non-attending women. This point has also been highlighted by other authors in the context of the Norwegian screening program [36].

The overall HPV positivity rate observed among women who attended screening using self-sampling was 13.1%, higher than the 9.9% recorded in the ordinary screening population in 2019 (*p* < 0.001). This higher HPV positivity rate can be attributed to a higher risk of HPV infection among non-attenders, with the highest positivity rate found in women who had never been screened, followed by those who had been screened more than 10 years ago and within 10 years (*p* = 0.000). As expected [38], HPV positivity rates were associated with women’s age, with the highest value observed in the 30–39 years age group (*p* = 0.027). Women tested mostly positive (65.7%) for the pool of 12 other high-risk HPV genotypes; no assessments regarding the prevalence of mono- or multi-HPV infections are possible, as full genotyping was not performed.

From a technical and organizational point of view, a low percentage of self-samples resulted inadequate (i.e., 1.1%), suggesting the ease of use of the collection method; data in the literature has reported rates of unsatisfactory self-collected samples ranging from 0.00% to 2.70% [31]. The rates of technically inadequate self-collected samples were associated with age, with the highest value observed in older woman (i.e., 2.1% in the 50–64 years age group); this association was also found by Ivanus and colleagues in the Slovenian cervical screening program [29]. In a very low percentage of cases, i.e., 0.5%, self-samples were not analyzed due to excessive delay in arriving at the laboratory, confirming the quality of the pathway involving local pharmacies.

The initial decision to adopt a direct referral to colposcopy policy for hrHPV positive women was based on data in the literature that showed an overall follow-up adherence nearly 22 percentage points higher in studies with direct referral compared to those with a cytology triage policy [31]. However, we found similarly high compliance rates for both immediate colposcopy and cytology triage on clinician-sample, i.e., >90% (*p* = 0.675), which is considered the desirable standard value in the document published by GISCi on indicators for monitoring screening programs using HPV testing for primary cervical cancer screening [39]. A high adherence to colposcopy after a positive cytology triage result was also observed, at 90.2%. Therefore, these results show that, in our setting, an additional visit to collect a cervical sample for cytological assessment did not compromise the completion of women’s screening pathway, but allowed us to avoid over-referral to colposcopy (about the 28% of women who underwent cytology triage had a positive result), which would have been a burden for the center in terms of organization and costs. In addition to the similar follow-up adherence observed when comparing the two triage policies, similar results were also obtained in terms of diagnostic yield. Indeed, although a higher overall PPV was obtained for cytology triage for CIN2+ in women who underwent colposcopy compared to HPV tests on self-collected samples, the difference was not significant (*p* = 0.400). However, the small sample size in the cytology triage group warrants caution when comparing diagnostic results between the two groups; moreover, a higher percentage of women were biopsied in the group referred directly to colposcopy.

Opposite to other authors who found higher rates of CIN2+ lesions in populations of non-attenders when the self-sampling method was offered [36,40], the present study observed no difference in the overall CIN2+ detection rate in the initiative target population compared to the value recorded in 2019 in the ordinary screening population (i.e, 8.5‰ versus 12.2‰, respectively; *p* = 0.147). Nevertheless, in the initiative target population, when compared with the ordinary screening population, slightly higher detection rates of CIN3 lesions were observed (i.e., 2.2‰ versus 1.5‰, respectively) and a cervical adenocarcinoma rate more than twice as high (i.e., 0.9‰ versus 0.4‰, respectively), reflecting an elevated risk of high-grade cervical lesions and cancer in underscreened women. Notably, the majority of CIN3+ diagnoses were recorded in women with no/low protection, suggesting a lack of effect from the organized screening in women who have never attended or did not attend screening for an extended period. In line with national data [41], the HPV genotype most frequently associated with high-grade cervical lesions and cancer was HPV16, accounting for 71.4% of cases.

None of the 12 women who received the HPV vaccine were diagnosed with CIN3+. Nevertheless, the very low incidence (i.e., 2%) of HPV vaccination in the study population and the mean age in which the third dose of vaccine was administrated do not allow for evaluations to be made. The observed incidence value of vaccination is attributed to the fact that the HPV vaccination campaign started in the Italian regions by the end of 2008, with 12-year-old pre-adolescent girls (born in 1997) as the primary target group [42].

Based on the effectiveness of offering self-sampling to reach underscreened women, the feasibility—regarding organization and costs—of routinely introducing the optional self-sampling method into the screening program for historically non-responding women is currently being evaluated at our center. The opportunity to offer the option of self-sampling, with an opt-in procedure, on the occasion of a screening participation reminder for women who did not attend the screening with clinical-sampling three years after the initial invitation is also under evaluation.

### Study Limits

This paper focuses on the adoption of the self-sampling method as a strategy to both resume cervical cancer screening activities disrupted by the COVID-19 pandemic and to reach underscreened women. The study specifically investigated the acceptance and ease of use of this method in a selected population of non-responders. Screening methodology was not addressed in this work. Issues related to the pre-analytical phase and laboratory processing of self-samples—such as the choice of swab type, sample elution medium type and volume, storage conditions, as well as the validation of specific cut-offs in HPV testing and specimen adequacy [15]—were not discussed, although these remain important open topics.

Additional limitations of the study include the small sample size of women in the cytology triage group classified as having “medium protection”, the very low percentage of HPV-vaccinated women in the study population, and the absence of full-genotyping.

## 5. Conclusions

Offering the self-sampling method as an option for women who never or irregularly attend the organized cervical cancer screening program of the LHA of Bologna, in addition to addressing the backlog caused by the COVID-19 pandemic, has proven to be an easy-to-use and effective tool for reaching these groups of women, who have been found to have an elevated risk for high-grade cervical lesions and cancer. Indeed, screening can identify precancerous lesions, allowing for timely treatment that prevents their progression to invasive cancer, avoiding the need for chemotherapy, radiotherapy, or the risk of infertility associated with cervix removal [43]. It has been estimated that organized screening has reduced cervical cancer rates by 50–80% [44]. Moreover, screening provides the best opportunity to diagnose cervical cancer at an early stage, when treatment is most effective. Irregular screening is an independent risk factor for late diagnosis [45]. When cervical cancer is diagnosed early, the 5-year relative survival rate is approximately 92%. Survival rates decline and the chance of recurrence increases for late diagnosis. If the cancer has spread locally or to regional lymph nodes at the presentation, the 5-year relative survival rate decreases to 60%. In cases where distant metastasis is present at diagnosis, the survival rate drops to 19% [45].

In our setting, the potential increase in screening coverage due to this optional collection method does not seem to be compromised by a cytology triage policy on clinician-collected samples following hrHPV positivity in self-samples.

Finally, our findings suggest the importance of maintaining the offer of clinician-collected screening for non-attenders.

## Figures and Tables

**Table 1:** Characteristics of the invited women.

Variable	Number	Percentage	Average Age in Years (Standard Deviation)
**Age group in year**			
30–39	8971	46.4%	34.8 (±2.8)
40–49	5352	27.7%	42.7 (±2.5)
50–64	5004	25.9%	59.4 (±3.0)
**Nationality**			
Italian	14,534	75.2%	43.9 (±10.6)
Other	4793	24.8%	41.8 (±9.6)
**District of residence**			
Pianura Ovest	1438	7.4%	41.5 (±9.0)
Città di Bologna	11,186	57.9%	42 (±9.9)
Pianura Est	2654	13.7%	42.7 (±10.0)
Reno, Lavino e Samoggia	1680	8.7%	49.2 (±11.3)
Savena Idice	1820	9.4%	45.9 (±11)
Appenino Bolognese	549	2.8%	52 (±10.9)
**Time since last screening test**			
Never screened (no protection)	12,280	63.5%	41.4 (±9.9)
≥10 years (low protection)	3393	17.6%	51.7 (±9.5)
<10 years (medium protection)	3654	18.9%	42.4 (±9.1)
**Total**	**19,327**	**100.0%**	**43.4 (±10.4)**

**Table 2:** Screening participation using self-sampling method, hrHPV-DNA positivity, and technically inadequate self-collected samples by demographic characteristics, district of residence, and screening attendance history. Bold *p*-values are statistically significant.

Variable	No.	Screening Participation by Using Self-Sampling Method No. (%)	*p*-Value	hrHPV-DNA Positivity No. (%)	*p*-Value	Inadequate Self-Collected Samples No. (%)	***p*** **-Value**
**Age group in years**							
30–39	8971	1091 (12.2%)	*p* = **0.010**	173 (15.9%)	*p* = **0.000**	11 (1.0%)	*p* = **0.021**
40–49	5352	612 (11.4%)		74 (12.1%)		2 (0.3%)	
50–64	5004	523 (10.54%)		44 (8.4%)		11 (2.1%)	
**Nationality**							
Italian	14,534	1818 (12.5%)	*p* = **0.000**	244 (13.4%)	*p* = 0.289	21 (1.2%)	*p* = 0.436
Other	4793	408 (8.5%)		47 (11.5%)		3 (0.7%)	
**District of residence**							
Pianura Ovest	1438	187 (13.0%)	*p* = **0.009**	27 (14.4%)	*p* = 0.719	0 (0.0%)	*p* = 0.590
Città di Bologna	11,186	1252 (11.2%)		170 (13.6%)		13 (1.0%)	
Pianura Est	2654	349 (13.2%)		47 (13.5%)		6 (1.7%)	
Reno, Lavino e Samoggia	1680	172 (10.2%)		17 (9.9%)		2 (1.2%)	
Savena Idice	1820	212 (11.7%)		24 (11.3%)		2 (0.9%)	
Appenino Bolognese	549	54 (9.8%)		6 (11.1%)		1 (1.9%)	
**Time since last screening test**							
Never screened (no protection)	12,280	1194 (9.7%)	*p* = **0.000**	176 (14.7%)	*p* = **0.027**	15 (1.3%)	*p* = 0.309
≥10 years (low protection)	3393	460 (13.6%)		56 (12.2%)		2 (0.4%)	
<10 years (medium protection)	3654	572 (15.7%)		59 (10.3%)		7 (1.2%)	
**Total**	**19,327**	**2226 (11.5%)**		**291 (13.1%)**		**24 (1.1%)**	

**Table 3:** Results of multivariate regression analysis of screening participation using the self-sampling method by demographic characteristics, district of residence, and screening attendance history. Bold *p*-values are statistically significant.

Variable	Odds Ratio	Standard Error	95% CI	*p*-Value
**Log likelihood = −6799.80, χ^2^ = 207.66 (10df), *p* < 0.00001, No. of obs = 19,327**				
**Age group in years**				
30–39	1.00 *			
40–49	0.84	0.05	0.76–0.94	**0.002**
50–64	0.72	0.05	0.64–0.82	**<0.001**
**Nationality**				
Italian	1.00 *			
Other	0.64	0.04	0.57–0.72	**<0.001**
**District of residence**				
Città di Bologna	1.00 *			
Pianura Ovest	1.07	0.09	0.91–1.26	0.424
Pianura Est	1.12	0.07	0.99–1.28	0.078
Reno, Lavino e Samoggia	0.92	0.08	0.78–1.09	0.324
Savena Idice	1.01	0.08	0.87–1.19	0.849
Appenino Bolognese	0.87	0.13	0.65–1.16	0.340
**Time since last screening test**				
Never screened (no protection)	1.00 *			
≥10 years (low protection)	1.61	0.10	1.42–1.82	**<0.001**
<10 years (medium protection)	1.74	0.10	1.56–1.95	**<0.001**

* Reference category.

**Table 4:** Women who tested positive for hrHPV-DNA: HPV genotypes and vaccine.

HPV Genotype	Number of HPV Genotype (%)		YES HPV Vaccine			Total Vaccine YES (%)
		NO HPV Vaccine (%)	Bivalent Against HPV 16, 18	Tetravalent Against HPV 16, 18, 6, 11	Nonavalent Against HPV 16, 18, 6, 11, 31, 33, 45, 52, 58	
HPV 16	50 (17.2)	48 (17.2)	0	1	1	2 (16.7)
HPV 16 and 18	3 (1.0)	3 (1.1)	0	0	0	0 (0.0)
HPV 16 and 18 and OHR *	2 (0.7)	2 (0.7)	0	0	0	0 (0.0)
HPV 16 and OHR	21 (7.2)	20 (7.2)	0	0	1	1 (8.3)
HPV 18	17 (5.8)	16 (5.7)	0	1	0	1 (8.3)
HPV 18 and OHR	7 (2.4)	7 (2.5)	0	0	0	0 (0.0)
OHR HPV	191 (65.7)	183 (65.6)	1	2	5	8 (66.7)
**Total**	**291**	**279 (95.9%)**	**1**	**4**	**7**	**12 (4.1%)**

* Other High-Risk. HPV 31, 33, 35, 39, 45, 51, 52, 56, 58, 59, 66, and 68 detected as a pool of HPV genotypes.

**Table 5:** Positive Predictive Value (PPV) of the HPV test on self-collected samples (a) and cytology triage on clinician-collected samples (b) for CIN2+ in women who underwent colposcopy.

**(a) Immediate Referral to Colposcopy**							
**Time Since Last Screening Test**	**No. of ** **Colposcopies**	**No. of ** **Biopsies**	CIN 2 *	**CIN 3 ^#^**	**Cervical ** **Adenocarcinoma ^¥^**	**CIN2+**	**PPV**
Never-screened women (no protection) ≥10 years (low protection)	95	11	5	3	1	9	9.5%
<10 years (medium protection)	23	48	3	1	0	4	17.4%
**Total**	**118**	**59 (50.0%)**	**8**	**4**	**1**	**13**	**11.0%**
**(b) Cytology Triage on Clinician-Sample**							
**Time Since Last Screening Test**	**No. of** **Colposcopies**	**No. of ** **Biopsies**	CIN 2 *	**CIN 3 ^#^**	**Cervical ** **Adenocarcinoma ^¥^**	**CIN2+**	**PPV**
Never-screened women (no protection) ≥10 years (low protection)	28	4	4	1	1	6	21.4%
<10 years (medium protection)	9	10	0	0	0	0	0.0%
**Total**	**37**	**14 (37.8%)**	**4**	**1**	**1**	**6**	**16.2%**

* HPV genotypes associated with the 12 CIN2 lesions: HPV 16 (n = 3; monoinfections), HPV16, HPV18 and OHR HPV (n = 1; multi-infection), HPV16 and OHR HPV (n = 2; multi-infections), and OHR HPV (n = 6; mono/multi-infections); ^#^ HPV genotypes associated with the 5 CIN3 lesions: HPV 16 (n = 3; monoinfections) and OHR HPV (n = 2; mono/multi-infections); ^¥^ HPV genotypes associated with the 2 cervical adenocarcinoma: HPV16 (n = 2; monoinfections).

## Data Availability

The original contributions presented in the study are included in the article, further inquiries can be directed to the corresponding author.
